# Supplementary material for: Improving foot self-care in people with diabetes in Ghana: A development and feasibility randomised trial of a context appropriate, family-orientated diabetic footcare intervention
Source: PLoS One. 2024 May 8;19(5):e0302385. doi: 10.1371/journal.pone.0302385 (PMC11078378; doi:10.1371/journal.pone.0302385)
Supplement: S2 File — (DOCX) [file pone.0302385.s002.docx]

**Family-oriented Diabetic Foot Self-care Programme in Ghana; A Feasibility Randomised Controlled Trial with nested qualitative interviews at the Komfo Anokye Teaching Hospital.**

**A Research Protocol**

**Submitted to the Food and Drugs Authority, Ghana**

**By**

**Joseph N. Suglo (PI) ……………………**

**Professor Jackie Sturt (First Supervisor) ……………………………**

**Dr Kirsty Winkley (Second supervisor) ……………**

1. Title: Family-oriented Diabetic Foot Self-care Programme in Ghana; A Feasibility Randomised Controlled Trial with nested qualitative interviews at the Komfo Anokye Teaching Hospital.

# Investigators:

1. Principal Investigators
2. Joseph Ngmenesegre Suglo (PI), PhD student, Florence Nightingale Faculty of Nursing, Midwifery and Palliative Care, King’s College London. [Joseph.suglo@kcl.ac.uk](mailto:Joseph.suglo@kcl.ac.uk)
3. Dr. Frank Botsi Micah (Co-PI), Head of Department for Internal Medicine, Komfo Anokye Teaching Hospital.
4. Supervisors:
5. Jackie Sturt, Professor of Behavioural Medicine in Nursing, Florence Nightingale Faculty of Nursing, Midwifery and Palliative Care, King’s College London. [Jackie.sturt@kcl.ac.uk](mailto:Jackie.sturt@kcl.ac.uk)
6. Kirsty Winkley (PhD), Diabetes Specialist Nurse, Reader in diabetes and Primary Care, Clinical Academic, Florence Nightingale Faculty of Nursing, Midwifery and Palliative Care, King’s College London. [Kirsty.winkley@kcl.ac.uk](mailto:Kirsty.winkley@kcl.ac.uk)
7. Research site: Diabetes Clinic, Komfo Anokye Teaching Hospital (KATH) – Ghana
8. **Sponsor:** Kings College London
9. Funder: Self-funded
10. Phase of trial: Not applicable
11. Trial monitor: Dr. Osei Sarfo-Kantanka (BSc, MBChB, FWACP (Endocrinology), Head of Diabetes Unit, Komfo Anokye Teaching Hospital. Email: [osarfokantanka21@gmail.com](mailto:osarfokantanka21@gmail.com)
12. Trial registry: PACTR202201708421484
13. Ethics**:** Komfo Anokye Teaching Hospital Institutional Review Board approval reference KATH IRB/AP/139/21 dated 22/12/2021
14. **Executive Summary**

**Background**

The most common and expensive to treat complication of diabetes is diabetic foot ulcer (DFU), and persons with diabetes have about 25% risk of developing foot ulcers and neuropathies in their lifetime. Informal caregivers, herein refers to as family caregivers (persons providing unpaid services at home) can play significant role in patients’ disease management and involving them in self-care interventions could lead to improve diabetic foot outcomes. DFU preventive training and education programmes engaging both persons with diabetes and their family caregivers in the USA, China, Indonesia, and other countries, are known to have resulted in improved foot self-care practices and reduced incidence of foot ulcers. However, it is not known if such family-oriented interventions apply or is feasible in Ghana.

**Aim**

The primary aim of this research is to evaluate the feasibility of conducting a randomised controlled trial to investigate the effectiveness of a hands-on skills training and education on foot self-care programme for persons with diabetes and their family caregivers in Ghana. The research question is ‘can the provision of a family-oriented foot self-care skills training and education intervention improve foot care behaviour, foot care self- efficacy, knowledge of diabetic foot and diabetes distress among persons with diabetes and their caregivers in Ghana?’

**Methods**

An existing intervention identified through literature review will be adapted for feasibility testing in Ghana based on the UK Medical Research Council Recommendations for Development, Implementation, and Evaluation of complex interventions. A feasibility RCT programme with three months duration will be conducted. Study participants will include adults with diabetes aged ≥18 years and their family caregivers recruited as dyads. Participants will be randomly assigned to the intervention or control group and quantitative data collected to address feasibility issues. Participants views and experiences about the intervention programme will be explored through a small group pre-pilot and intervention-refinement study that will precede the feasibility trial. The pre-pilot phase will also assess the acceptability of the intervention in this population and incorporate any suggestions into the final adapted intervention.

**Full Protocol**

# Background rational of the study

Open wounds due to diabetes are the most common and expensive to treat complication of diabetes. Persons with diabetes have about 25% risk of developing these foot ulcers and neuropathies in their lifetime (Boulton, 2015; Singh et al., 2005). Epidemiological studies of foot ulceration in Ghana are limited. However, a multicentre cross-sectional survey in three major tertiary hospitals in Ghana indicated the prevalence of diabetic foot ulcers (DFU) among persons with diabetes on admission was 11% (Atosona & Larbie, 2019). Similarly, a 12-year retrospective study involving over 7300 persons with diabetes at one diabetes clinic in southern Ghana identified the incidence of DFU had increased from 3.25% in 2005 to 12.57% in 2016 (Sarfo-Kantanka et al., 2018). Even though the methodology may differ, these figures are considered alarming when compared with a DFU prevalence rate of 1.2%, 4.6%, 2.05% and 3.3% in Egypt, Kenya, Jordan, and Saudi Arabia respectively (Al-Rubeaan et al., 2015; Bakri et al., 2011; El-Nahas et al., 2008; Nyamu et al., 2004).

Informal caregivers, herein refers to as family caregivers (persons providing unpaid services at home) can play significant role in patients’ disease management and involving them in self-care interventions could lead to improve diabetic foot outcomes (Gilliss et al., 2019; Grey et al., 2015; Isworo et al., 2018; Messenger et al., 2019) Diabetic foot ulcer preventive education and training programmes engaging both persons with diabetes and their family caregivers in the USA, China, Indonesia, and other countries, are known to have resulted in improved diabetes knowledge, improved foot self-care practice and reduced incidence of foot ulcers. Despite increasing prevalence of diabetic foot ulcers in Ghana, such foot care interventions are yet to be tested or implemented in Ghana. For instance, a systematic review of the evidence preceding this study did not find any eligible informal caregivers intervention study from Ghana and Africa as a whole, and it remains unclear whether such interventions actively engaging both patient and family members will be effective and/or applicable in the Ghanaian health system. Nevertheless, based on the context and favourable outcomes, existing foot care intervention components were identified, and these could be applicable and potentially beneficial to Ghana. The active components of these interventions include engaging both caregivers and the person with diabetes in (1) Self-management activities (hands-on workshops on foot self-care and skills exercises) and (2) Family management (strengthening the family support on problem solving in DFU prevention, establishing family roles and effective involvement in DFU prevention) delivered through face-to-face intensive health education and skills training (Liang et al., 2012; Subrata et al., 2020). This existing intervention will be adapted for feasibility testing in Ghana. Therefore, this protocol describes a mixed method feasibility randomised controlled trial (RCT) of a Foot Selfcare Training and Education programme in Ghana. The intervention development/adaptation is guided by the Medical Research Council and the National Institute for Health Research (MRC-NIHR) framework for developing and evaluating complex interventions (Skivington et al., 2021), and underpinned by the Self-efficacy theory (Bandura, 1997). The study methods are discussed according to the Standard Protocol Items: Recommendations for International Trials (SPIRIT) guidelines and all elements of the SPIRIT checklist are reported (Chan et al., 2013).

# Aims and Objectives of the Study

The primary aim of this research is to evaluate the feasibility of conducting a randomised controlled trial to investigate the effectiveness of a hands-on skills training and education on foot self-care programme for persons with diabetes and their family caregivers in Ghana. The research question is ‘can the provision of a family-oriented foot self-care skills training and education intervention improve foot care behaviour, foot care self- efficacy, knowledge of diabetic foot and diabetes distress among persons with diabetes and their caregivers in Ghana?’

## 12a. Objectives of the study:

1. To modify and/or adapt the foot care programme through, making it context appropriate for the Ghanaian setting.
2. To explore the experiences and views of persons at high risk of diabetic foot ulcer, their family caregivers, and nurses on the foot self-care programme to facilitate further modification of the intervention and design of the trial.
3. To ascertain the acceptability of such family-oriented intervention in Ghana
4. To determine the feasibility of conducting a definitive trial in evaluating the effectiveness of family-oriented diabetic foot self-care intervention in Ghana.
5. To identify signals of efficacy of the intervention on various outcomes at post intervention

# Methods and Study Design

## 13a. **Theoretical underpinning and Framework of the Study**

- **Theoretical background:** Social Cognitive Theory (SCT) will be used to guide this intervention development and delivery because of its self-efficacy and environment constructs (Bandura 1997). The concept of the ‘environment’ in SCT is significant to this study as it embodies the persons around and interacting with the patient. Thus, the environment in this theory includes family members, friends, and other non-kin members within the social context of the person with diabetes. Self-efficacy is a component of Bandura’s SCT, which is a social learning theory. Bandura’s social cognitive theory advocates the use of demonstration in diabetic foot care instruction. Demonstrating foot care can increase self-efficacy in foot care behaviours by providing both an active mastery and vicarious experience. According to Bandura, people learn through observation due to the effects of imitation (Bandura 1997). The modelling effect, which includes the acquisition of novel responses, can be applied to the learning of foot care behaviours when nurses show foot care and patients return with a foot care demonstration. Furthermore, verbal persuasion by healthcare professionals can also influence foot care behaviours. Previous studies have demonstrated that training patients with diabetes can enhance foot care practices (Sloan 2002). These elements of the self-efficacy theory directly underpin the various components of the intervention.
- **Study Framework**: The UK Medical Research Council and National Institute of Health Research (MRC-NIHR) framework for development of complex interventions provides a flexible, less linear and robust process for developing or adapting existing complex interventions (Skivington et al., 2021). Therefore, this study will be guided by the recommendations of the MRC-NIHR framework. Even though this study will not be developing an intervention from the base, it will involve substantial modification to an existing intervention through stakeholder inputs and consultations. The study will be based on the first two phases of the framework: development (adaptation) and feasibility testing of a foot selfcare training and education programme. Guided by the MRC-NIHR framework, the study is being structured into phases as described below

13b. **Identifying the evidence**: This phase as recommended by the MRC-NIHR framework seeks to explore the literature for interventions in development or use for potential refinement. This has been completed and the aim was to: (1) determine the effects of interventions involving informal caregivers in the prevention and/or management of diabetic foot ulcers in adults (2) understand if combinations of intervention components were associated with particular foot ulcer clinical outcomes (3) explore potential refinement to existing relevant foot care interventions (Suglo et al 2021 manuscript under review). Based on the context and favourable intervention outcomes, this review of the literature has identified a foot selfcare intervention components used in Indonesia (Subrata et al., 2020) and China (Liang et al., 2012), and these will be adapted for feasibility testing in Ghana. (See page 17 & 18 of this document for detail description of the intervention).

13c. **Adaptation of the intervention and stakeholder group:** A foot selfcare training and education intervention identified from review of the evidence(Liang et al., 2012; Subrata et al., 2020) will be modified to be culturally relevant to the Ghana context. This will be done through stakeholder consultations. This initial intervention adaptation will be done by a purposively formed stakeholder group consisting of two diabetes nurses, a diabetes physician, two persons with diabetes as patients and two-family caregivers. In a meeting with the stakeholder group, the principal investigator will present the study/project and the proposed intervention then get comments. The stakeholders’ opinions and recommendations will be incorporated into adapting the intervention.

**13d. Pre-pilot and intervention refinement study (Study 1):** This phase, hereafter refer to as ‘Study 1’ is a nonrandomised one arm study to aid refine the intervention for the next phase of the study. In this study 1, five dyads (persons with diabetes and caregivers) and five nurses will be recruited, and all will receive the adapted intervention as one group. The intervention will be delivered to participants by trained providers who will be delivering the intervention during the pilot feasibility RCT. All participants in this study will be required to complete a set of questionnaires (Appendices 35) at least a day before the intervention. Individual interviews will also be conducted within the first one week after the intervention. The aim is to assess participants appraisal of the adapted questionnaires and intervention components for further refinement. Therefore, Study 1 has its own objectives as indicated below (10e). Eligibility criteria for participants and outcome measures for this study is same as that for the pilot feasibility RCT in the next phase. Following the completion of study 1, data will be analysed, and recommendations of participants will be considered before recruitment for the pilot feasibility RCT. The principal investigator will complete a Template for Intervention Description and Replication (TiDieR) checklist (Hoffmann et al., 2016) and document the rational for any necessary adaptations that occurred after study 1.

### **Specific objectives of study 1**

### **Persons with diabetes and Family Caregivers**

1. Explore their views on the content and delivery of the foot care training programme.
2. Examine participants understanding of the questionnaires used.
3. Explore the experiences of being a caregiver to someone with diabetes (**Caregivers only**)

### **For Nurse Participants**

1. Assess the nurses’ views on the content of the foot care training programme
2. Examine from the nurses’ perspective the barriers and facilitators to delivering foot care training programme in the hospital.

**13e. Pilot Feasibility RCT (Study 2):** This study hereafter called ‘Study 2’ will be a parallel two arm randomised pilot feasibility study (n = 50) conducted after completion of Study 1. The aim at this stage is to assess trial procedures or feasibility issues and explore potential effects of the foot care programme**.** This will include assessment of recruitment and retention of participants, acceptability of randomisation process, data completion and signals of intervention efficacy. Data capturing forms and questionnaires (See appendices) will be used to obtain information from both the intervention group and control group. Figure below gives a summary of the methodology and processes of this study.

**
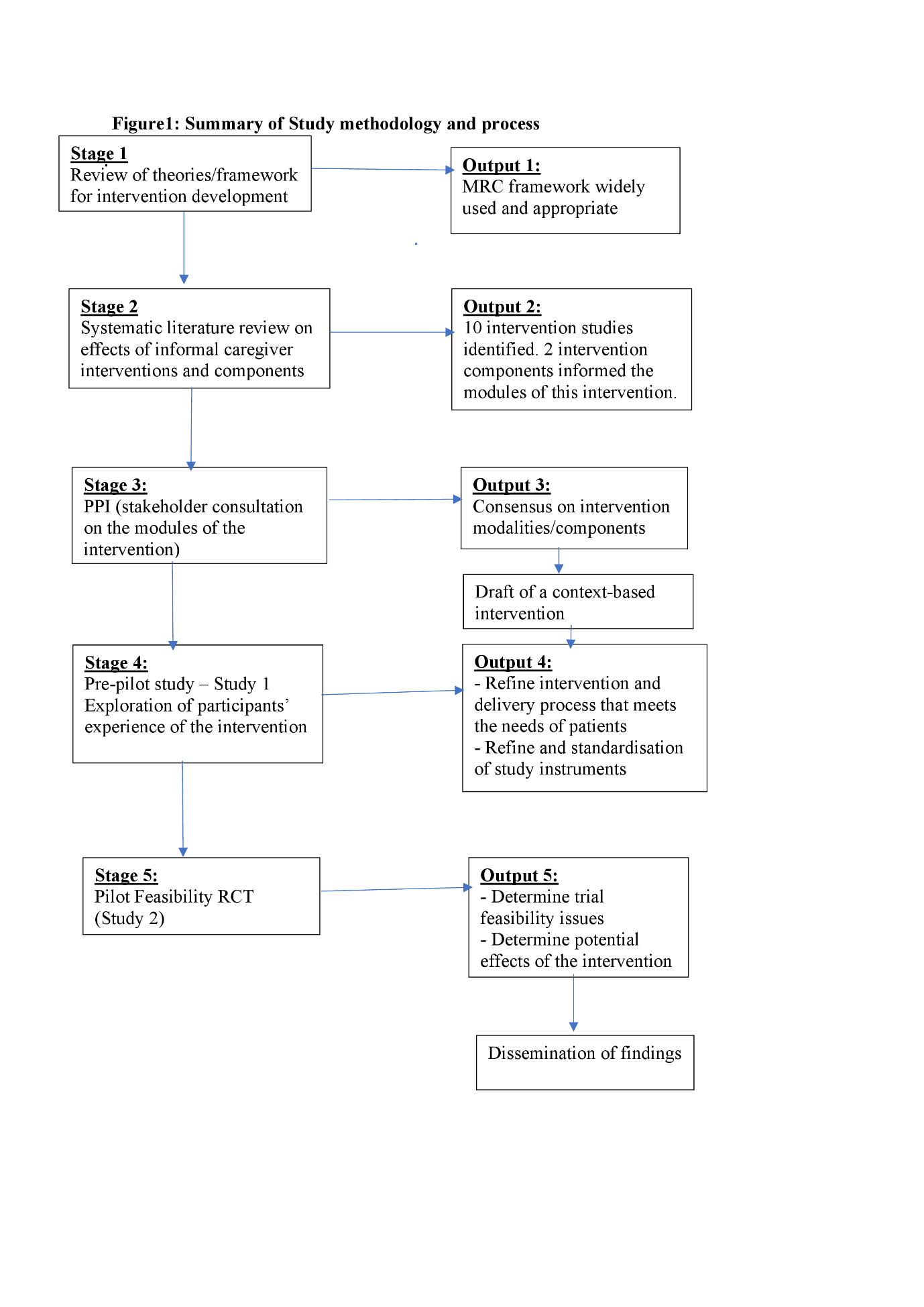
**

# Study Setting

The study site will be the diabetes clinic of Komfo Anokye Teaching Hospital (KATH), Kumasi – Ghana. KATH is centrally located in Kumasi, the administrative capital of the Ashanti region of Ghana. The region has a population of about 5,432,485 (Ghana Statistical Service 2021) and because of its central location in the country, it receives referral patients from at least 12 out of the 16 administrative regions of Ghana and from neighbouring countries such as Burkina Faso and Ivory Coast (KATH, 2020). Published statistics indicates that the diabetes clinic from 2012 to mid-year of 2019 has a total registered patient population of about 83,361 (KATH, 2020). It is a public hospital providing services to Ghanaians of all age categories and social classes. As a teaching/tertiary hospital, majority of patients have received diabetes care at the primary care level before being referred to seek further care at KATH. Conducting the study at KATH is deemed appropriate because of its large diverse patient population, staff skill mix, strategic location and being one of the biggest of the six teaching/tertiary hospitals in Ghana.

# Study Population

The study will recruit persons with diabetes, caregivers and nurses as described below.

(**a). Patient Participants**: The study population will consist of persons with pre-existing diagnosis of type 1 or 2 diabetes who attended a scheduled review at the diabetes clinic of the Komfo Anokye Teaching Hospital (KATH), Kumasi - Ghana. Persons with diabetes will participate in the study with an invited family caregiver (Dyad). These persons will be recruited for both Study 1 and Study 2.

**(b) Nurse Participants**: Nurses who are registered with the nurses and midwifery Council of Ghana and are currently working at a diabetes clinic will participate in Study 1.

# 16. Participant’s inclusion and exclusion criteria

Recruitment for the various categories of participants will be based on the following criterion:

## Inclusion Criteria for Persons with Diabetes

- Adults aged ≥18 years with a confirmed diagnosis of type 1 or 2 diabetes
- Participants fulfil at least one of the following three criteria for definition of high risk for foot ulcers; (i) medically confirmed diagnosis of neuropathy indicated in records, (ii) previous/healed diabetic foot ulcer or (iii) foot abnormalities at risk of ulcer in the opinion of the investigator (iv) venous insufficiency (skin colour change or temperature difference) (ADA 2018).
- Have a family caregiver who live with them and/or assisted them in their day-to-day self-care activities and willing to participate in the study with them.
- Persons who attended the KATH diabetes review clinic or were admitted to the KATH diabetes unit.
- Able to provide informed consent to participate in the trial.

## Inclusion criteria for family caregivers

- Persons ≥18 years and living with the individual with diabetes and/or assisted them on daily basis with care activities.
- Individuals willing to participate in the study and able to provide an informed consent.

## Exclusion criteria for persons with diabetes and caregivers

- People with peripheral vascular disease requiring immediate revascularization.
- Persons with severe mental illness or cognitive impairment
- Persons with current foot ulcers.
- Persons who participated in the pre-pilot study and interviews that preceded the feasibility RCT.

# Sample Size

The study will recruit a total number of 115 participants for both study 1 and study 2. This will comprise of 55 dyads (persons with diabetes and their caregivers) and 5 nurses working in the diabetes clinic. Sample size for the various phases of the study is based on the aim of the study and pragmatic reasons as indicated below.

**a. Sample size for Study 1**: In qualitative and feasibility studies, sample size may be determined based on the objectives of the study and resource availability (Baker & Edwards, 2012; Britten, 1995). Since this phase is meant to obtain opinions for the refinement of the intervention for a feasibility trial, and based on resources and time constraints, only a sample size of 5 dyads and 5 nurses will be recruited using purposive sampling technique.

b. **Sample size for Study 2:** Feasibility trials are not aimed at establishing an intervention’s superiority but rather to test procedures, processes and help in sample size calculation of the definitive trial by getting estimates of the parameters (Arain et al., 2010; Thabane et al., 2010). Therefore, it is suggested that feasibility studies do not need power-based sample size calculation (Arain et al., 2010; Lancaster et al., 2004; Whitehead et al., 2016). Sample size in part is determined by the amount of variance in the outcomes of interest in the population (Thabane et al., 2010). It is generally considered that a sample size of 50 participants is adequate to determine this variability. Considering that this is a feasibility trial, the study will recruit 100 individuals (50dyads) with 25dyads in each arm of the study.

# 18. Recruitment and Consent Process

**a. Patients and Caregivers**: Participants will be recruited from the diabetes clinic of KATH where persons with diabetes are reviewed by the attending physicians and nurses. Posters inviting persons with diabetes to the study and the contact details of the principal investigator will be displayed on main notice boards and consulting rooms. The support of the clinic nurses will be solicited so that they give verbal reminders and invitations to prospective participants on every review day. Potential participants will be screened to determine their eligibility to participate in the study with the help of clinic staff. Persons with diabetes wishing to participate in the study will also be required to invite a family caregiver who usually assist them with care and is willing to participate in the study. Persons who are eligible and wish to participate will then be directed to the principal investigator for further information. The research process will then be explained in detail to each eligible participant. They will also be provided with an information sheet and consent form. Separate consent forms and information sheets will be prepared for participants in Study 1 and Study 2. Participants will be asked to read the given information sheets and determine whether they wish to participate and proceed to sign the consent form. Participants who cannot sign will be required to thumb print and indicate their consent for participation.

**b. Nurse Participants**: Nurses will be recruited only for the study 1, and this will be done purposefully so that nurses who have worked extensively with persons with diabetes at the clinic will be recruited. Nurses at the clinic will be approached by the principal investigator and the nature of the study explained to them. Persons who are willing to participate will be provided with further details using the information sheet and subsequently provision of a signed consent form voluntarily by participants.

# 19. **Randomisation, Allocation Concealment and Blinding**

- **Randomisation**: The eligible dyad participants will randomly be assigned in a 1:1 ratio to one of two groups (group I as intervention group and group II as control group) using a remote computer-generated random number sequence by an independent statistician from the Kintampo Health Research Centre (KHRC). Upon recruiting an eligible dyad participant, a telephone call or text message will be sent to the statistician for random allocation of the dyad. Group allocation will be communicated to participants by the principal investigator and intervention sessions will start within one week after getting a group of at least 12 dyad participants.
- **Allocation Concealment:** Participants upon signing the study consent will complete the questionnaires as part of their baseline assessments before randomisation to ensure concealment of the allocation sequence. The statistician will generate and keep the randomisation list. Thus, the principal investigator, research nurses and other personnel involved in recruitment or outcome assessment will not have access to the randomisation list.
- **Blinding**: Due to the physical nature of the intervention, participants, the principal investigator, and providers delivering the intervention will not be blinded. However, outcomes assessors collecting follow up data will be blinded to group allocation.

# 20. Participants withdrawal from the study

Participants will be informed in the participant information sheet that after they provide consent, they can withdraw at any time if they want. Because this is a feasibility study, the researcher would like to know the reasons for withdrawal to help plan the definitive trial. However, participants will not be obliged to provide reasons for withdrawal if they do not want to. Participants may no longer be able to withdraw their data from the study after the analysis of the data because it would have been anonymised and analysed. (See dates indicated in information sheets).

# 21. Outcomes

All questionnaires/data capturing forms will be in English language but will be linguistically and culturally adapted to the setting in accordance with the guidelines described by Wild and colleagues (Wild et al., 2005). English language is the official language use in Ghana, and participants who do not understand English language will have the study documents read and verbally translated into the native language to them. To assess feasibility issues and investigate potential effects of the intervention, the following outcomes will be measured. Some outcomes are for both the person with diabetes and caregivers, and others are for only persons with diabetes as indicated below.

**All Participants**

- **Demographic Characteristics:** The demographic characteristics of persons with diabetes consisting of age, gender, marital status, educational level, duration of diabetes, diabetes type, treatment method and receipt of diabetes education will be collected. Caregiver information including gender, age, relationship with patient, time range being a caregiver and education level will be recorded. Collection of these information will be conducted at baseline to be able to know persons who have participated in the study and the possible recruitment procedures to follow for a definitive study.
- **Feasibility outcomes**: The primary evidence sought through this study is to determine the feasibility of conducting a definitive trial that will assess the effectiveness of a foot self-care programme in Ghana. To assess feasibility issues, data will be recorded pertaining to recruitment, withdrawal, and completion of data. This will be done using a screening and enrolment form designed by the principal investigator. Thus, data on the number of persons screened, eligible participants who were recruited (i.e. provided consent to be randomised into the study), participants who completed the intervention period and those who completed all assessments will be captured. The following cut-off points will be used to determine feasibility: recruitment rate of 50% (of participants screened as eligible for the study) and completion rate of 70% (including all assessments, intervention, and follow-up periods).
- **Diabetic foot Knowledge**: Foot self-care knowledge will be measured using the diabetes knowledge questionnaire (DKQ-24), which was developed by Garcia and colleagues (Garcia et al., 2001). The DKQ is made of 24 questions with 5-items relating specifically to knowledge on diabetic foot. The questionnaire developed and validated in both English and Spanish languages has a coefficient α of 0.78 with coefficients ranging from 0.73 to 0.84 for all three subgroups - participants with diabetes, support for people with diabetes, and nondiabetic support persons. Even though the DKQ-24 has a relatively lower coefficient (α of 0.78) compared with the original 60 item questionnaire (DKQ-60) with a coefficient of 0.83, the instrument demonstrated internal consistency and construct validity, and is easy to use for persons with lower-level literacy (Garcia et al., 2001). The comparatively lower reliability coefficient may be due to the length of the test.

**For Persons with Diabetes Only**

- **Foot Care behaviour of participants**: The foot care practices of participants will be assessed using an adapted version of the Nottingham Assessment of Functional Footcare (NAFF) (Lincoln et al, 2007). NAFF is a 29 item self-reported measure of the extent to which people comply with recommended foot care behaviours. The development of this questionnaire initially involved 100 persons with diabetes and 61 healthy adults resulting in a 51-item scale. This was shortened to 29 items with an internal consistency of 0.53. The test-retest reliability was also found to be good (Lincoln et al, 2007). The NAFF was used in a randomized controlled trial (Lincoln et al, 2008) to assess the effectiveness of education in foot ulcer prevention and was found to be sensitive to the effects of the educational intervention. The NAFF could be used to identify compliance with recommended foot care practice in routine clinical practice. The NAFF validity was assessed again involving 115 persons and this resulted in a Cronbach’s alpha reliability of 0.61 for the 29-item scale. Also, the Pearson’s correlation between test and re-test was 0.92 (p < 0.001) indicating the NAFF was internally consistent and reliable overtime (Senussi et al 2011)
- **Foot self-care efficacy:** This outcome measures how confident the participants feel in undertaking foot self-care practices. The Foot Care Confidence Scale (FCCS) (Sloan, 2002) measures self-confidence of persons with diabetes in caring for their feet using 12 items. The FCCS developed and validated among diverse American populations and settings, had a Cronbach’s alpha of 0.92. Factor analysis revealed that all 12 items loaded on one factor and all 12 items are needed to measure confidence to care for one’s feet. The FCCS is a practical questionnaire for use in many settings, especially where time constraints exist, and its 5-point scale makes it suitable for persons with low literacy skills (Sloan, 2002). The questionnaire has since been effectively used to measure patient’s self-efficacy in many RCTs and varying settings (Keller-Senn et al., 2015; Mahdalena & Ningsih, 2016; Nguyen et al., 2019).
- **Foot Problems**: Participants will self-report any changes in foot problems such as cuts, blisters, or lesions on their feet since the start of the intervention programme. Both control and study group participants will also have their feet inspected at baseline and follow-up to determine if there have been any changes from baseline. The outcome assessor will call participants monthly on phone to check if they have developed any foot problems. Any identified or reported foot problems will be recorded using a data capturing form designed by the researcher.

**For Family Caregivers Only**

- **Diabetes Distress**: Diabetes distress (DD) affects caregivers’ quality of life and not only persons with diabetes (Polonsky et al., 2016). Therefore, interventions might be better sustained, and their effects maximised if they reduce caregivers DD. Family caregivers will be assessed using the Diabetes Distress Scale for Spouses and Partners (DDS-SP) (Polonsky et al., 2016). The questionnaire contains 22-items categorized into four subscales labelled as Hypoglycaemia Distress (four items), Emotional Distress (five items), Management Distress (seven items) and Role Distress (five items). The DDS-SP has a good internal consistency (Cronbach’s α = 0.95), and all subscales have a Cronbach’s α ranging from 0.85-0.93). This questionairre measures distress that is more specific and related to diabetes. Previous studies have successfully used the DD scale to assess distress among family members and friends of persons with diabetes (Berry et al., 2018).

# 22. **Data collection**

- **For study 1:** Following the signing of informed consent, the principal investigator will collect baseline data using the questionnaires and other data capturing forms before the delivery of the intervention. These will be completed by the participants at least a day before the intervention delivery. All questionnaires will be printed and handed in paper format. Within a week after the intervention, individual interviews will be conducted with all participants either on a face-to-face basis at the diabetes clinic or on telephone depending on preference of the participant. An interview guide will be developed based on review of the literature and consideration of the objectives of the study. The interview guide will have three major sections, one each for persons with diabetes, caregivers, and nurses. The data collection interviews will be conducted in English language or in a native Ghanaian language for persons who cannot speak English language at a location and time convenient to participants and the researcher. Informed consent will be obtained to audio record all interviews. Audio recordings will be done using SONY ICD PX333 Digital Voice Recorder. A mobile phone will also be used as a secondary option for recording should the former have problems.
- **For Study 2**: Data for the pilot feasibility RCT will be collected at baseline and follow up data at 12 weeks post baseline. The baseline assessment data will be done by the principal investigator while a trained nurse researcher blinded to group allocation of participants will collect the follow up data. This will be done during the medical appointment at the diabetes review clinic or at the participant’s home. However, if covid19 pandemic persist and face-to-face data collection is not possible, it will be done via mobile phone, in such cases outcomes like feet inspection for foot problems will be self-reported by participants and not objectively by the assessor. The contact numbers of participants and caregivers will be collected to facilitate follow up. At the end of each follow up visits, the assessor will be asked if he had been ‘unblinded’ and if not to make a guess whether a participant belongs to intervention or control groups. This forms part of feasibility assessment to ascertain if masked outcome assessment is possible in a definitive trial.

# 23. **Data Management and Monitoring**

Each participant will have an assessment form completed at baseline with assistance from the researcher and at post intervention by a research nurse trained for this purpose. All participants will be assigned a trial identification number (ID) and same on all questionnaires or documents used to collect data. An electronic database will be created using Microsoft Access Software to input and store data collected. Data collected with the questionnaires and other source documents will be inputted to this database, password protected and securely handled by the principal investigator in an assigned university laptop. Missing data will be minimised by maintaining contact with participants during the study period. Each returned questionnaire will be checked for missing responses and if incomplete, the principal investigator will contact participants by telephone or wait for them to come for review for the missing information to be collected. The data will then be ‘transported’ to the Statistical Package for Social Sciences (SPSS) software for data cleaning and analysis. At least 50% of data will be double entered for accuracy before analysis.

# 24. **Data Analysis**

- **For study 1:** The audio recordings of individual interviews will be played, and verbatim transcription done. If some participants opt to be interviewed in any other language, their interviews will be transcribed and translated to English to enhance accuracy of the reported data (Squires 2008). Data will then be analysed thematically guided by the approach described by Braun and Clarke (Braun & Clarke, 2006). Thematic analysis as described by Braun and Clarke has six stages which include familiarization with text through reading, generation of initial codes in a systematic fashion, collating codes into possible themes, reviewing of themes, clear definition, and naming of themes and writing-up of final analysis. The final analysis will be written with supportive verbatim quotation to answer the specific objectives of the study. The process is recursive and non-linear, and this gives the opportunity to move back and forth between stages as may be necessary. This analysis approach is useful for exploring the experiences of different participants, identifying their similarities, differences and gaining detail understanding of the data (Braun & Clarke, 2006). Questionnaires administered will be entered in SPSS software to determine their Cronbach’s alpha coefficient. Rewording of outcome measures will be done in areas that were not clear or understood by participants.
- **For Study 2:** Quantitative data collected with the questionnaires will be entered into the Statistical Package for Social Sciences version 22 for analysis. Descriptive statistics will be used to describe participants demographic characteristics and other variables. Means and standard deviations of participants Knowledge, foot care behaviour, foot selfcare efficacy and diabetes distress, will be computed using their scores at baseline and follow-up. The adjusted and unadjusted mean differences (95% CI) will also be computed. Frequencies and percentages for participants characteristics, foot problems, recruitment, retention, and data completion rates will be calculated and presented in tables.

# 25. Standard of care for diabetic patients for which the intervention will be trialed against.

The diabetes clinic at the Komfo Anokye Teaching Hospital (KATH) operates four clinic days in a week for persons with diabetes. Attendance is by clinic appointment given to patients who were either referred from other facilities or were diagnosed of diabetes at KATH. During such visits, patients are monitored for their Temperature, Blood Pressure, Weight and Random Blood sugar level by the attending nurses. The patient then goes into the consulting room of the physician for medical assessment and treatment. It should be noted that patients are not mandated or instructed to attend the clinic with their family caregiver(s); education on diabetes and footcare is only provided on ad-hoc basis and there is currently no information leaflet/booklet to guide how such information should be provided. Thus, education on foot selfcare and diabetes in general is not structured and not part of the routine practice at the clinic. Foot care skills training engaging both the patient and caregiver is non-existent at the clinic. Also, apart from information leaflets on healthy nutrition, patients are not provided with any information materials for reference at home.

# **26. The Intervention**

The intervention is a nurse-led ‘Foot Selfcare Training and Education’ programme using basic skills to empower patients take care of their own feet through daily inspection. The intervention integrates self-efficacy enhancing activities and education and has been designed to enable persons with diabetes and their caregivers to gain confidence and skills and in caring for their feet. Components of this intervention include (1) Self-management (hands-on workshops on foot self-care and education) and (2) Family management (strengthening the family support on problem solving in diabetic foot prevention, establishing family roles and effective involvement in foot ulcer prevention) delivered through face-to-face intensive health education and skills training. Participants in the intervention group will each receive (i) nail clippers for proper cutting of toenails (ii) a plastic handy mirror for inspection of bottom of feet, (iii) bag to contain all equipment. After self-management education, demonstration by the nurse and return demonstration of foot care by patients and caregivers, each participant, assisted by their caregiver will be asked to perform daily foot care at home. Detail of the intervention session plan presented in table 1 below. Guided by the evidence from a systematic review, the revised intervention from study 1 will be delivered in four 50-minutes sessions over four weeks.

Table 1: Intervention programme sessions for patients and caregivers

| Week | Topic/duration | Content of sessions | Components of self-efficacy learning being use and delivery methods |
| --- | --- | --- | --- |
| **1** | Topics:   1. Diabetes and the Diabetic foot 2. Family Caregiver roles and support in foot care   Duration: 50 minutes | - Programme introduction (overview) - Diabetes education (definition, causes/risk factors, treatment and self-management, diabetes as a manageable chronic disease, overview of complications) - Providing information on how the feet is affected by diabetes. - Teaching on awareness of serious foot problems - Family caregiver role in prevention of foot problems - Discussion and teaching on caregiver roles and responsibilities - Discussion facilitators and barriers amid family involvement in foot ulcer prevention - Discussion problem-solving strategies to deal with foot problems. - Motivating caregiver to actively support in foot check activities | Intensive health education  Verbal persuasion  Peer learning |
| **2** | Topic:   1. Diabetic footcare for the individual   Duration: 50 minutes | - Recap of family caregiver roles from the previous session. - Teaching on daily foot self-care activities and monitoring: - Daily foot inspection for foot problems - Daily washing and drying of feet, use of moisturiser - Massaging foot and foot exercise - Footwear - Nail care and when to seek care from a health professional. - Annual foot examination by health professional | Intensive health education  Verbal persuasion and encouragement  Identifying and adjusting to stress and harnessing the support of social environment (caregiver)  Problem-solving  Peer learning |
| **3** | Topic:  Experiential workshop, skills training on footcare  Duration: 50 minutes | Skills demonstrating and role play on how to carry out foot checks at home:   - Physical feet inspection for problems detection - Use of mirrors to check bottom of feet, - Use of proper footwear - Use of nail clippers to cut nails straight across - Use of 10g monofilament to check for protective feet sensation - Return demonstrating of skills by study participants | Mastery experiences  Vicarious learning  Verbal encouragement, and feedback.  Skills training/Discussion |
| **4** | Topic: Reinforcement of foot education and skills training  Duration 50 minutes | - Recap of the various foot care activities taught in week 2 - Skill demonstrating and role play of various skills learnt in week 3 - Questions and answer session to clarify any doubt. - Refreshment of participants to conclude the programme | Skills training/Discussion  Vicarious learning/verbal encouragement |

# 27.

# **Intervention provider training and intervention fidelity**

Two research nurses with experience of working in a diabetes clinic and similar patient population will deliver the intervention at an identified location within KATH. They will undergo a one-day training in facilitation skills associated with the diabetes foot selfcare programme. This will include practical skill development and underlying theoretical principles of self-efficacy. The research nurse training syllabus will provide an experiential learning for nurses by incorporating efficacy enhancing mechanisms. Thus, syllabus of the training for intervention providers will include (i) self-efficacy theory and its components (Positive verbal persuasion/encouragement; positive vicarious learning; personal mastery; adjustment to stress and the social environment) (ii) Structure/components of the intervention programme and (iii) practical skill development in foot care (See appendices for the content of training programme for nurses delivering the intervention). Trained research nurses will deliver the intervention during study 1, and it will serve as avenue to further upskill themselves in the intervention delivery procedures for study 2. The nurse training programme will be accompanied by detail written materials to which the nurse can refer during the intervention delivery. This will assure that a standardised delivery of the intervention is carried out. To further assess intervention providers adherence to the programme, a checklist assessing the incorporation of each specific efficacy-enhancing techniques and coverage of all intervention components will be used by the principal investigator to assess the delivery process in at least 50% of the intervention sessions.

# **28. Control Situation**

Both intervention and control groups will continue to receive the standard care (usual care) as described earlier under section 25. The control group will receive only usual care during this study. Usual care refers to the health care rendered to persons who attended the diabetic clinic for their medical or health appointments. This care may include but not limited to health assessment and/or treatment rendered by nurses and physicians to patients seeking health care services at the diabetic clinic. Thus, participants of this study will not be stopped from receiving their standard care from the hospital. Within a week after collecting follow-up data from the control group, the foot selfcare intervention will be offered to participants in a group by the principal investigator at a venue within the diabetes clinic.

# 29. Covid19 Contingency Plan

The practical nature of the intervention (skills training workshop) coupled with likely poor internet connectivity in Ghana might not support the intervention delivery over the telephone or other video calls. Therefore, if there is a new covid19 wave, groups will further be divided into smaller units that allows for the observance of social distancing protocols during the delivery of the intervention. However, all outcome assessments will be conducted through telephone calls or prepaid post letters. In such covid19 situations, observation of feet for foot problems will be self-reported by participants instead of objective assessment by the assessor.

# 30. **Ethical consideration**

Ethical approval for both study 1 and 2 will be sought from King’s College Research Ethics Committee and Komfo Anokye Teaching Hospital Institutional Review Board- Ghana. The study will also be registered with the Research and Development Unit of KATH in accordance with the local research governance policy. The principal investigator will obtain participants consent using a written consent form, and a contact for redress will be provided. Participant’s confidentiality and data protection for all participants will be ensured as discussed in the next section. To avoid disadvantaging participants in the control group, the intervention education materials and other potential benefits will be presented to them at the end of follow up data collection. Thus, within a week after follow-up data collection has completed, the principal investigator will arrange a group meeting with all members in the control group. At this meeting, education on foot care, using the intervention materials will be delivered to all participants. Also, persons with diabetes who were willing but could not participate in the study because of unavailable family caregiver will be informed to contact the principal investigator for the intervention materials at the end of the study. They will also be signposted to other online foot care resources and identified personnel at the study site they could contact for support with their foot care needs.

# 31. Confidentiality and Anonymity

Each participant’s personal details will be linked to their study identifier in an excel spreadsheet that is password protected that only members of the research team will have access to. Data will be processed in accordance with the General Data Protection Regulation 2016 (GDPR). King’s College London (KCL) is the sponsor for this study based in the United Kingdom. KCL will keep identifiable information about participants for 7 years after the study has completed and afterward data will be destroyed permanently.

Participants names will be removed and replaced with a code, and they will not be identified in any of the information use during the study or when results are shared. Depersonalized direct quotes will be included in this study. This will also be published in relevant journals and presented as part of the dissemination strategy. No name will appear in any reports that are generated from this study. Only the research team will have access to the identifiable information and the entire interview transcripts. Audio-recordings will be stored electronically on KCL network and will be encrypted, and password protected. The answered questionnaires will be anonymized. Personal information such as contact details, and consent forms will be kept separately from depersonalised data, filed, and kept in a locked cabinet in the researcher’s office at the King’s College London. For the purposes of good clinical practice (GCP) audit or inspection, the ethics committee, Food and Drugs Authority, monitors and auditors may be granted direct access to participants information.

# 32. Benefits and Potential Beneficiaries

Persons with diabetes and their caregivers who participate in this study will help to refine and develop a context specific diabetic foot selfcare management programme for persons with diabetes. They may benefit from foot selfcare skills and information needs when this foot care programme is developed and hopefully integrated into the Ghanaian health care system. This study cannot guarantee that all participants will personally benefit from the research findings, but all persons with diabetes and caregivers will benefit in the future when the evidence generated from this study begins to shape the design and delivery of foot care programmes. Also, nurses (Providers delivering the intervention) will be upskilled through training in the foot care programme.

# 33. Incentives for Participants

Each dyad participants will be reimbursed with 20 Ghana cedis as their transportation cost since the intervention days might be outside of their routine clinic review schedule. This token will be given after each intervention session. Also, the footcare materials provided to participants for the study will be left with them for use at home. Thus, participants will be allowed to keep the mirrors, 10g monofilaments, nail clippers and bags that were provided as part of the intervention. This will facilitate continues practice of skills that were acquired from the study.

# 34. Dissemination

Two mains peer reviewed papers are expected to be developed from this study for publication in Open Access Journals. The first will be presenting the study protocol and the target journal will be the BMC journal for Pilot and Feasibility Studies. Another research paper will be expected from the intervention modification process and feasibility trial. We will aim to publish this in Diabetic Medicine journal. The International Diabetes Federation (IDF) congress will be targeted for conference presentations and discussion of our project processes and outcomes. Links to published results, study protocol, and summary of the trial results will also be made available on the Pan African Clinical Trials Registry.

# 35. Duration of the Study

It is estimated that the entire study duration, from the commencement of study 1 to completion of study 2 will be seven (7) months. The table and consort diagram below gives some further details of the sequence of planned activities

# Timelines

| Research Activity | Timeline |
| --- | --- |
| Development of research materials (consent forms, information sheets, intervention manual) | October – December 2021 |
| R & D registration and submission for ethics | October – December 2021 |
| Pre-pilot and intervention refinement (Study 1) | January – March 2022 |
| Data Analysis for Study 1 | March- April 2022 |
| Participants recruitment and intervention delivery for feasibility RCT (Study 2) | April – June 2022 |
| Data collection | July – September 2022 |
| Data analysis for study 2 | October – December 2022 |
| Writing of Results and Discussion | January – June 2023 |
| Publications and Submission of Thesis | July – September 2023 |

# Figure 1: Consort diagram for participants in feasibility trial

Eligibility assessment by attending physicians, nurses, and researcher

Enrolments – week 1 - 4

Exclusion

- Do not meet inclusion criteria
- Decline to participate
- Other reasons

Initial approach by attending physicians and nurses

Informed consent by the principal investigator

Baseline assessments by the principal investigator

Randomisation by an independent statistician using a computer-generated system

Intervention delivery – weeks 5 - 8

Diabetic foot selfcare training and education intervention 50 minutes weekly for 4 weeks

By trained nurses, plus usual care by diabetic unit

Usual care

By diabetes clinic review nurses and physicians

Follow-up at weeks 16

Follow-up assessment at two months (8 weeks) by outcome assessor

# 36. **Trial Management**

The trial will be managed by the principal investigator (JNS) and his academic supervisors, meeting monthly via Microsoft Teams during the period of intervention delivery and data collection. JNS will be responsible for the day-to-day administration of the study at the study site and reporting to the academic supervisors through emails and monthly supervision meetings. All documents will be prepared and kept at the study site. Two nurses will be trained to deliver the intervention and a third nurse, blinded to participant group allocation will be trained to collect the follow-up data. The principal investigator will be responsible for the recruitment, random sequence allocation of study participants, data entry and analysis. An independent statistician at an identified research centre (Kintampo Health Research Centre) will be responsible for generating and keeping the randomisation list.

# 37. **Personnel of the Study**

1. Mr. Joseph Ngmenesegre Suglo; Principal Investigator (PI)
2. Dr. Frank Botsi Micah (Co-PI)
3. Professor Jackie Sturt; First Supervisor
4. Dr. Kirsty Winkley; Second Supervisor

# 38. Reference

Al-Rubeaan, K., Al Derwish, M., Ouizi, S., Youssef, A. M., Subhani, S. N., Ibrahim, H. M., & Alamri, B. N. (2015). Diabetic foot complications and their risk factors from a large retrospective cohort study. *PLoS ONE*, *10*(5). https://doi.org/10.1371/JOURNAL.PONE.0124446

Arain, M., Campbell, M. J., Cooper, C. L., & Lancaster, G. A. (2010). What is a pilot or feasibility study? A review of current practice and editorial policy. *BMC Medical Research Methodology*, *10*(1), 67. https://doi.org/10.1186/1471-2288-10-67

Atosona, A., & Larbie, C. (2019). Prevalence and Determinants of Diabetic Foot Ulcers and Lower Extremity Amputations in Three Selected Tertiary Hospitals in Ghana. *Journal of Diabetes Research*, *2019*. https://doi.org/10.1155/2019/7132861

Baker, S. E., & Edwards, R. (2012). *How many qualitative interviews is enough*.

Bakri, F., Allan, A., Khader, Y., … N. Y.-J. M., & 2012, undefined. (2011). Prevalence of diabetic foot ulcer and its associated risk factors among diabetic patients in Jordan. *Platform.Almanhal.Com*. https://platform.almanhal.com/Files/2/36282

Bandura, A. (1997). *Self-Efficacy: The Exercise of Control* . Freeman and Company. https://books.google.co.uk/books/about/Self_Efficacy.html?id=eJ-PN9g_o-EC

Berry, E., Davies, M., & Dempster, M. (2018). Managing Type 2 diabetes as a couple: The influence of partners’ beliefs on diabetes distress over time. *Diabetes Research and Clinical Practice*, *141*, 244–255. https://doi.org/10.1016/J.DIABRES.2018.05.020

Boulton, A. J. M. (2015). The diabetic foot. *Medicine*, *43*(1), 33–37. https://doi.org/10.1016/j.mpmed.2014.10.006

Braun, V., & Clarke, V. (2006). Using thematic analysis in psychology. *Qualitative Research in Psychology*, *3*(2), 77–101. https://doi.org/10.1191/1478088706QP063OA

Britten, N. (1995). Qualitative Research: Qualitative interviews in medical research. *BMJ*, *311*(6999), 251–253. https://doi.org/10.1136/BMJ.311.6999.251

Chan, A. W., Tetzlaff, J. M., Altman, D. G., Laupacis, A., Gøtzsche, P. C., Krleža-Jerić, K., Hróbjartsson, A., Mann, H., Dickersin, K., Berlin, J. A., Doré, C. J., Parulekar, W. R., Summerskill, W. S. M., Groves, T., Schulz, K. F., Sox, H. C., Rockhold, F. W., Rennie, D., & Moher, D. (2013). SPIRIT 2013 statement: Defining standard protocol items for clinical trials. *Annals of Internal Medicine*, *158*(3), 200–207. https://doi.org/10.7326/0003-4819-158-3-201302050-00583

El-Nahas, M. R., Gawish, H. M. S., Tarshoby, M. M., State, O. I., & Boulton, A. J. M. (2008). The prevalence of risk factors for foot ulceration in Egyptian diabetic patients. *Practical Diabetes International*, *25*(9), 362–366. https://doi.org/10.1002/PDI.1311

Garcia, A. A., Villagomez, E. T., Brown, S. A., Kouzekanani, K., & Hanis, C. L. (2001). The Starr County Diabetes Education Study. *Diabetes Care*, *24*(1), 16–21. https://doi.org/10.2337/DIACARE.24.1.16

Gilliss, C. L., Pan, W., & Davis, L. L. (2019). Family Involvement in Adult Chronic Disease Care: Reviewing the Systematic Reviews. *Journal of Family Nursing*, *25*(1), 3–27. https://doi.org/10.1177/1074840718822365

Grey, M., Schulman-Green, D., Knafl, K., & Reynolds, N. R. (2015). A revised Self- and Family Management Framework. *Nursing Outlook*, *63*(2), 162–170. https://doi.org/10.1016/j.outlook.2014.10.003

Hoffmann, T. C., Glasziou, P. P., Boutron, I., Milne, R., Perera, R., Moher, D., Altman, D. G., Barbour, V., MacDonald, H., Johnston, M., Lamb, S. E., Dixon-Woods, M., McCulloch, P., Wyatt, J. C., Chan Phelan, A. W., Michie, S., & Voigt-Radloff, S. (2016). Better Reporting of Interventions: Template for Intervention Description and Replication (TIDieR) Checklist and Guide. *Gesundheitswesen*, *78*(3), 175–188. https://doi.org/10.1055/s-0041-111066

Isworo, A., Ekowati, W., Iskandar, A., & Latifah, L. (2018). Family Involvement Programmes on the Metabolic Response of Diabetic Patients. *Health Science Journal* , *12*(2), 1–3. https://search.proquest.com/openview/2f59e8ac560568883091385726a81a3f/1?pq-origsite=gscholar&cbl=237822

KATH, K. (2020). *About Us | Komfo Anokye Teaching Hospital*. http://www.kathhsp.org/about-us/

Keller-Senn, A., Probst, S., Imhof, R. M., & Imhof, L. (2015). Nurse-led education programme enhancing foot care self-efficacy in high-risk diabetes population: pilot randomised controlled study. *Http://Dx.Doi.Org/10.1179/2057331615Z.0000000009*, *12*(2), 74–78. https://doi.org/10.1179/2057331615Z.0000000009

Lancaster, G. A., Dodd, S., & Williamson, P. R. (2004). Design and analysis of pilot studies: Recommendations for good practice. *Journal of Evaluation in Clinical Practice*, *10*(2), 307–312. https://doi.org/10.1111/j..2002.384.doc.x

Liang, R., Dai, X., Zuojie, L., Zhou, A., & Meijuan, C. (2012). Two-Year Foot Care Program for Minority Patients with Type 2 Diabetes Mellitus of Zhuang Tribe in Guangxi, China. *Canadian Journal of Diabetes*, *36*(1 CC-Wounds), 15‐18. https://doi.org/10.1016/j.jcjd.2011.08.002

Mahdalena, M., & Ningsih, E. S. P. (2016). Effectivity of Foot Care Education Program in Improving Knowledge, Self-Efficacy and Foot Care Behavior among Diabetes Mellitus Patients in Banjarbaru, Indonesia. *Kesmas: Jurnal Kesehatan Masyarakat Nasional (National Public Health Journal)*, *11*(2), 56–60. https://doi.org/10.21109/KESMAS.V11I2.583

Messenger, G., Taha, N., Sabau, S., AlHubail, A., & Aldibbiat, A. M. (2019). Is There a Role for Informal Caregivers in the Management of Diabetic Foot Ulcers? A Narrative Review. *Diabetes Therapy*, *10*(6), 2025–2033. https://doi.org/10.1007/s13300-019-00694-z

Nguyen, T. P. L., Edwards, H., Do, T. N. D., & Finlayson, K. (2019). Effectiveness of a theory-based foot care education program (3STEPFUN) in improving foot self-care behaviours and foot risk factors for ulceration in people with type 2 diabetes. *Diabetes Research and Clinical Practice*, *152*, 29–38. https://doi.org/10.1016/J.DIABRES.2019.05.003

Nyamu, P. N., Otieno, C. F., Amayo, E. O., & Mcligeyo, S. O. (2004). Risk factors and prevalence of diabetic foot ulcers at Kenyatta National Hospital, Nairobi. *East African Medical Journal*, *80*(1), 36–43. https://doi.org/10.4314/eamj.v80i1.8664

Polonsky, W. H., Fisher, L., Hessler, D., & Johnson, N. (2016). Emotional distress in the partners of type 1 diabetes adults: Worries about hypoglycemia and other key concerns. *Diabetes Technology and Therapeutics*, *18*(5), 292–297. https://doi.org/10.1089/DIA.2015.0451

Sarfo-Kantanka, O., Kyei, I., Mbanya, J. C., & Owusu-Ansah, M. (2018). Diabetes-related foot disorders among adult Ghanaians. *Diabetic Foot and Ankle*, *9*(1). https://doi.org/10.1080/2000625X.2018.1511678

Singh, N., Armstrong, D., & Benjamin, L. (2005). Preventing foot ulcers in patients with diabetes. *Jama*, *293*(2), 217–228. https://jamanetwork.com/journals/jama/article-abstract/200119

Skivington, K., Matthews, L., Simpson, S. A., Craig, P., Baird, J., Blazeby, J. M., Boyd, K. A., Craig, N., French, D. P., McIntosh, E., Petticrew, M., Rycroft-Malone, J., White, M., & Moore, L. (2021). A new framework for developing and evaluating complex interventions: update of Medical Research Council guidance. *BMJ*, *374*, n2061. https://doi.org/10.1136/BMJ.N2061

Sloan, H. L. (2002). Developing and testing of the foot care confidence scale. *Journal of Nursing Measurement*, *10*(3), 207–218. https://doi.org/10.1891/jnum.10.3.207.52564

Subrata, S. A., Phuphaibul, R., Grey, M., Siripitayakunkit, A., & Piaseu, N. (2020). Improving clinical outcomes of diabetic foot ulcers by the 3-month self- and family management support programs in Indonesia: A randomized controlled trial study. *Diabetes and Metabolic Syndrome: Clinical Research and Reviews*, *14*(5), 857–863. https://doi.org/10.1016/j.dsx.2020.05.028

Thabane, L., Ma, J., Chu, R., Cheng, J., Ismaila, A., Rios, L. P., Robson, R., Thabane, M., Giangregorio, L., & Goldsmith, C. H. (2010). A tutorial on pilot studies: The what, why and how. *BMC Medical Research Methodology*, *10*(1), 1–10. https://doi.org/10.1186/1471-2288-10-1

Whitehead, A. L., Julious, S. A., Cooper, C. L., & Campbell, M. J. (2016). Estimating the sample size for a pilot randomised trial to minimise the overall trial sample size for the external pilot and main trial for a continuous outcome variable. *Statistical Methods in Medical Research*, *25*(3), 1057–1073. https://doi.org/10.1177/0962280215588241

Wild, D., Grove, A., Martin, M., Eremenco, S., McElroy, S., Verjee-Lorenz, A., & Erikson, P. (2005). Principles of Good Practice for the Translation and Cultural Adaptation Process for Patient-Reported Outcomes (PRO) Measures: report of the ISPOR Task Force for Translation and Cultural Adaptation. *Value in Health : The Journal of the International Society for Pharmacoeconomics and Outcomes Research*, *8*(2), 94–104. https://doi.org/10.1111/J.1524-4733.2005.04054.X

# 39. Appendices

# QUESTIONAIRE BOOKLET FOR PERSONS WITH DIABETES

Date: -----------------------------------------------

Participant Study ID ------------------------------

This booklet is made of four parts: A-D, comprising of Demographic information, Diabetic foot Knowledge, foot care behaviour and foot care confidence scale respectively. Please read and answer all sections.

# Part A: Demographic Characteristics

The questions below seek your demographics information and your diabetes. Please answer each question by ticking (√) the box that corresponds to your answer.

| 1 | **What is your gender?** |  |
| --- | --- | --- |
|  | Male |  |
|  | Female |  |
|  | Prefer not to answer |  |
|  |  |  |
| 2 | **What is your marital status?** |  |
|  | Married |  |
|  | Single |  |
|  | Other (Specify)…………….. |  |
|  |  |  |
| 3 | **Your highest educational level attained?** |  |
|  | Have not being to school |  |
|  | Primary school |  |
|  | Junior secondary school/O-Level |  |
|  | Senior secondary/senior |  |
|  | Tertiary |  |
|  |  |  |
| 4 | **Your diagnosed diabetes type:** |  |
|  | Type 1 |  |
|  | Type 2 |  |
|  | Do not know |  |
|  |  |  |
| 5 | **What Type of treatment are you currently taking?** |  |
|  | Diet |  |
|  | Insulin |  |
|  | Non-insulin injectable (GLP1-RA) |  |
|  | Oral hypoglycaemics agents |  |
|  | Insulin, oral hypoglycaemic agents |  |
|  |  |  |
| 6 | **How long have you been living with diabetes (in years)?** |  |
|  | Less than 5 years |  |
|  | 5 – 10 years |  |
|  | 11 – 15 years |  |
|  | 16-20 years |  |
|  | 21 and above |  |

# Part B: Diabetes Knowledge Questionnaire-24 (DKQ-24)-Diabetes Foot Knowledge Questionnaire

Read each of the following statement about diabetic foot and indicate your response by ticking (√).

|  | **Yes** | **No** | **I don’t know** |
| --- | --- | --- | --- |
| 1. Diabetes often causes poor circulation |  |  | **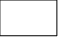** |
| 1. Cuts and abrasions on diabetes heal more slowly |  |  | **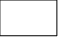** |
| 1. People with Diabetes should take extra care when cutting their toenails |  |  |  |
| 1. A person with diabetes should cleanse a cut with iodine and alcohol |  |  |  |
| 1. Diabetes can cause loss of feeling in my hands, finger, and feet |  | 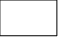 | **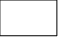** |
| 1. Tight elastic hose or socks are not harmful for diabetics. | 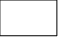 |  | **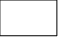** |
| **Total score** | **0–6** |  |  |

# Part C: Foot Care Behaviour (Nottingham Assessment of Functional Footcare)

We would like to know what you do to look after your feet. Please tick (√) the category which best reflects **what you actually do**. Please answer all questions. Thank you.

| 1 | **How often do you examine your feet?** |
| --- | --- |
|  | Once a day  Once a week  or less  2-6 times  a week  More than  once a day |
| 2 | **Do you check your shoes before you put them on?** |
|  | Often  Never  Rarely  Sometimes |
| 3 | **Do you check your shoes when you take them off?** |
|  | Often  Never  Rarely  Sometimes |
| 4 | **Do you wash your feet**? |
|  | Most days  a week  A few days/never  a week  Once a day  More than  once a day |
| 5 | **Do you check your feet are dry after washing?** |
|  | Rarely  Sometimes  Never  Often |
| 6 | **Do you dry between your toes?** |
|  | Always  Often  Somethings  Rarely/Never |
| 7 | **Do you use moisturising cream on your feet?** |
|  | About once a month  Once a week  Never  Daily |
| 8 | **Do you put moisturising cream between your toes?** |
|  | Daily  About once a month  Never  About once a week |
| 9 | **How often do you cut your toenails?** |
|  | Less than once a month  About once a month  Never  About once a week |
| 10 | **Do you wear slippers with no fastening?** |
|  | Rarely  Never  Most of the time  Sometimes |
| 11 | **Do you wear trainers?** |
|  | Never  Rarely  Most of the time  Sometimes |
| 12 | **Do you wear shoes with lace-up, Velcro or strap fastenings?** |
|  | Sometimes  Rarely  Never  Most of the time |
| 13 | **Do you wear pointed-toed shoes?** |
|  | Never  Rarely  Sometimes  Most of the time |
| 14 | **Do you wear open sandals/Slippers?** |
|  | Rarely  Sometimes  Never  Most of the time |
| 15 | **Do you put on your new shoes slowly and gradually?** |
|  | Most of the time  Always  Rarely /Never  Sometimes |
| 16 | **Do you wear artificial fibre (e.g. nylon) socks?** |
|  | Rarely  Never  Sometimes  Most of the time |
| 17 | **Do you wear shoes without socks/stockings/tights?** |
|  | Often  Sometimes  Rarely  Never |
| 18 | **How often do you change your socks/stockings/tights?** |
|  | 19  4-6 times  a week  Less than  4 times a week  Daily  More than  once a day |
| 19 | **Do you walk around the house in bare feet?** |
|  | Sometimes  Never  Rarely  Often |
| 20 | **Do you walk outside in bare feet?** |
|  | Often  Never  Rarely  Sometimes |
| 21 | **Do you use a hot water bottle in bed?** |
|  | Rarely  Sometimes  Never  Often |
| 22 | **Do you put your feet near the fire?** |
|  | Sometimes  Rarely  Never  Often |
| 23 | **Do you use corn remedies/corn plasters/ paints when you get a corn?** |
|  | Sometimes  Often  Rarely  Never |
| 24 | **Do you put a dry dressing on a blister when you get one?** |
|  | Sometimes  Often  Rarely  Never |
| 25 | **Do you put a dry dressing on a bruise, cut or burn when you get one?** |
|  | Sometimes  Often  Rarely  Never |

# Part D: Foot Care Confidence Scale (FCCS)

The following questions ask about how confident you feel performing foot care. For each item, circle the number that gives the best answer for you. Please provide an answer for each question.

To what level do you feel you can perform the following foot care activities? **Key**: Strongly lack confidence (1), moderately lack confidence (2), confident (3), moderately confident (4) and strongly confident (5).

|  | Strongly lack confidence | Moderately lack confidence | confident | Moderately confident | Strongly confident |
| --- | --- | --- | --- | --- | --- |
| 1. I am able to inspect and protect my feet | 1 | 2 | 3 | 4 | 5 |
| 1. I can look at my feet daily to check for cuts, scratches, blisters, redness or dryness | 1 | 2 | 3 | 4 | 5 |
| 1. I can dry between my toes after washing my feet | 1 | 2 | 3 | 4 | 5 |
| 1. I can judge when my toenails need to be trimmed by a podiatrist | 1 | 2 | 3 | 4 | 5 |
| 1. I can trim my toenails straight across | 1 | 2 | 3 | 4 | 5 |
| 1. I can figure out when to use a pumice stone to smooth corns and/or calluses on my feet | 1 | 2 | 3 | 4 | 5 |
| 1. I can test the temperature of the water before putting my feet into it | 1 | 2 | 3 | 4 | 5 |
| 1. I can wear shoes and socks every time I walk (includes walking indoors) | 1 | 2 | 3 | 4 | 5 |
| 1. When I go shopping for new shoes, I can choose shoes that are good for my feet | 1 | 2 | 3 | 4 | 5 |
| 1. I can call my doctor about problems with my feet | 1 | 2 | 3 | 4 | 5 |
| 1. Before putting them on, I can check the insides of my shoes for problems that could harm my feet | 1 | 2 | 3 | 4 | 5 |
| 1. If directed to do so, I can routinely apply lotion to my feet | 1 | 2 | 3 | 4 | 5 |

**THANK YOU FOR TAKING PART**

# QUESTIONAIRRE BOOKLET FOR CAREGIVERS

Date: -----------------------------------------------

Participant Study ID ------------------------------

This booklet is made of three sections: A-C, comprising of Demographic information, Diabetic foot Knowledge, and diabetes distress respectively. Please read and answer all sections.

# Section A: Demographic Characteristics of Caregiver

**The following questions relates to your demographic information. Please indicate your response by ticking (√) the box that corresponds to your chosen answer.**

| 1 | **What is your gender?** |  |
| --- | --- | --- |
|  | Male |  |
|  | Female |  |
|  | Prefer not to answer |  |
|  |  |  |
| 2 | **What is your marital status?** |  |
|  | Married |  |
|  | Single |  |
|  | Other (Specify) ………… |  |
|  |  |  |
| 3 | **Your highest educational level attained?** |  |
|  | Have not being to school |  |
|  | Primary school |  |
|  | Junior secondary school/O-Level |  |
|  | Senior secondary/senior |  |
|  | Tertiary |  |
|  |  |  |
| 4 | **What is your relationship with the patient** |  |
|  | Partner/Spouse |  |
|  | Parent |  |
|  | Son/daughter |  |
|  | Other (Specify) |  |
|  |  |  |
| 5 | **How long have you been a caregiver?** |  |
|  | Less than 5 years |  |
|  | 5-10years |  |
|  | Above 10 years |  |

# Section B: Diabetes Knowledge Questionnaire-24 (DKQ-24)-Diabetes Foot Knowledge Questionnaire

Read each of the following statement about diabetes and foot complications and indicate your response by ticking (√).

|  | **Yes** | **No** | **I don’t know** |
| --- | --- | --- | --- |
| 1. Diabetes often causes poor circulation |  |  | **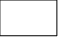** |
| 1. Cuts and abrasions on diabetes heal more slowly |  |  | **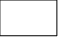** |
| 1. People with Diabetes should take extra care when cutting their toenails |  |  |  |
| 1. A person with diabetes should cleanse a cut with iodine and alcohol |  |  |  |
| 1. Diabetes can cause loss of feeling in my hands, finger, and feet |  | 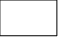 | **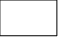** |
| 1. Tight elastic hose or socks are not harmful for diabetics. | 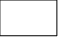 |  |  |
| **Total score** | **0–6** |  |  |

# Section C: Caregiver or Partner Diabetes Distress Scale

The following questions ask about how you have been feeling as a caregiver or partner of someone with diabetes. For each item, **circle the number** that gives the best answer for you, indicating 0 – not all, 1 – a little, 2 – somewhat, 3 – a lot, 4 – a great deal. Please provide an answer for each question.

**During the past month, I have been**:

|  | Not at all | A little | Somewhat | A lot | A great deal |
| --- | --- | --- | --- | --- | --- |
| 1. Worrying about my family member’s low blood sugars. | 0 | 1 | 2 | 3 | 4 |
| 1. Feeling unclear about exactly how much I should be involved in managing his/her diabetes. | 0 | 1 | 2 | 3 | 4 |
| 1. Frustrated that my family member shuts me out of his or her diabetes | 0 | 1 | 2 | 3 | 4 |
| 1. Feeling that my family member doesn’t try hard enough to manage his/her diabetes. | 0 | 1 | 2 | 3 | 4 |
| 1. Feeling overwhelmed by the constant demands of his or her diabetes. | 0 | 1 | 2 | 3 | 4 |
| 1. Worrying that I don’t know how to best help him or her manage diabetes. | 0 | 1 | 2 | 3 | 4 |
| 1. Feeling that I stay silent about his or her diabetes more than I really should. | 0 | 1 | 2 | 3 | 4 |
| 1. Feeling that diabetes is taking up too much of my mental and physical energy every day. | 0 | 1 | 2 | 3 | 4 |
| 1. Feeling that no one notices that diabetes is hard on me, not just on my family member. | 0 | 1 | 2 | 3 | 4 |
| 1. Frustrated that the more I try to help him or her manage his/her diabetes, the worse things get between us. | 0 | 1 | 2 | 3 | 4 |
| 1. Feeling guilty about not doing enough to help my family member with diabetes | 0 | 1 | 2 | 3 | 4 |
| 1. Frustrated that I can’t get my family member to improve his/her attitude about diabetes. | 0 | 1 | 2 | 3 | 4 |
| 1. Worrying that I am failing to help my partner or family member manage diabetes more successfully. | 0 | 1 | 2 | 3 | 4 |
| 1. Feeling that trying to help my family member or partner with his/her diabetes is always a battle. | 0 | 1 | 2 | 3 | 4 |
| 1. Frustrated because s/he ignores my suggestions about diabetes. | 0 | 1 | 2 | 3 | 4 |
| 1. Frustrated that diabetes often interrupts our plans. | 0 | 1 | 2 | 3 | 4 |
| 1. Worrying about my partner’s or family member’s low blood sugars when he/she is sleeping. | 0 | 1 | 2 | 3 | 4 |
| 1. Worrying about him or her driving because of possible low blood sugars. | 0 | 1 | 2 | 3 | 4 |
| 1. Worrying about leaving him or her alone because of the possible danger of low blood sugars | 0 | 1 | 2 | 3 | 4 |
| 1. Concerned that me and him/her are not working well together when it comes to diabetes. | 0 | 1 | 2 | 3 | 4 |
| 1. Feeling that I never get a break from worrying about his or her diabetes. | 0 | 1 | 2 | 3 | 4 |

**THANK YOU FOR PARTICIPATING**

# Interview Topic Guide for Study 1

**Instructions**

- Explain the objectives of this study to interviewee
- Get consent for the interview and audio recording
- The interview guide is divided into three sections; A, B and C. Section A is for participants with diabetes, section B is for caregivers of persons with diabetes and section C is for Nurse participants. Use the appropriate section for each participant.

**Interview topic Guide**

**Section A: For participants with diabetes**

1. Please tell me about your feelings/impressions about the foot care training and education programme? **Prompts:** components (Content, duration, delivery process by the nurse, timing, engagement/participation, venue, resources/logistics, applicability in everyday life)
2. Please tell me about what you have understood from the training and education programme? **Prompts:** selfcare (foot care, knowledge/awareness about foot disease, care skills). How different is it from what you knew?
3. What outcomes or benefits will you expect to/likely to get from this and similar foot care programmes? What do you consider as important to you in prevention of foot disease? **Prompts:** awareness or knowledge on …, skills, behaviour, peer or family supports?
4. Generally, what will you say about the questionnaires/outcomes that you completed **Prompts:** understanding (language, clarity), significance or relevance to you, appropriateness?
5. How will you describe your expectations in relation to this programme? **Prompts**: Satisfaction with…, willingness to participate in similar future programmes, recommendation for others?
6. Anything else you want to say about this programme and future research?

**End of Interview for patients**

**Section B: For Family Caregiver Participants**

1. Please tell me about a typical day in terms of being a caregiver. **Prompts**: Diet planning/cooking, assisting with diabetes care, workload, stress and worries, difficulties, facilitators
2. Please tell me about your feelings/impressions about the foot care training and education programme received? Prompts: components (Content, duration, delivery process by the nurse, timing, engagement/participation, venue, resources/logistics, applicability in everyday life)
3. Generally, what will you say about the questionnaires/outcomes that you completed? **Prompts:** understanding (language, clarity), significance or relevance to you or appropriateness?
4. How will you describe your expectations in relation to this programme? **Prompts**: Satisfaction with…, willingness to participate in such future programmes, recommendation for others?
5. What do you think about being a caregiver? **Prompts**: Lifestyle changes/ tell me more about that, anything else? How do you feel about that?
6. Anything else you want to say about this programme and future research?

**End of Interview for caregivers**

**Section C: Nurse participants**

1. Please tell me about your feelings/impressions about the foot care training and education programme? Prompts: components (Content, duration, delivery process by the nurse, timing, engagement/participation, venue, resources/logistics, applicability in everyday life)
2. What do you think about organising this and similar foot care training/education programme in your practice area/hospital? Prompts: Facilitators/enablers, barriers/challenges
3. Anything else you want to say about this programme and future research?

**End of Interview for Nurses**

**THANK YOU FOR PARTICIPATING**
