# Supplementary material for: Improving foot self-care in people with diabetes in Ghana: A development and feasibility randomised trial of a context appropriate, family-orientated diabetic footcare intervention
Source: PLoS One. 2024 May 8;19(5):e0302385. doi: 10.1371/journal.pone.0302385 (PMC11078378; doi:10.1371/journal.pone.0302385)
Supplement: S3 File — (DOCX) [file pone.0302385.s003.docx]

**Membership and composition of PPI group**

| **Stakeholder** | **Relevant experience** | **Expected role/contribution** |
| --- | --- | --- |
| Two persons with diabetes | Persons with diabetes that have been accessing diabetes care for at least five years | - Review intervention components presented to them. - Contribute by writing or verbally to aspects of intervention that should be modified or added to make the intervention context relevant. - Comment on the relevance and practicality of the intervention content/components in the context - Comment on appropriateness and relevance of patient reported outcome measure instruments. - Comment on any other thing they deem important to the intervention in the context |
| Two Family caregivers, not necessarily related to the other stakeholder with diabetes | Informal carers for persons with diabetes for at least five years |  |
| Two registered nurses | Nurses with contextual understanding of issues and provision of health care services to patients in the study context for over five years |  |
| One physician | A medical doctor with contextual understanding of issues and provision of health care services to persons with diabetes in this context for over five years |  |
| One academic (lecturer) | A researcher with previous experience of recruiting stroke survivors and their family caregivers into a study |  |
| One person from patient advocate group (Ghana Diabetes Association) | This is the only Ghanaian diabetes association recognised by IDF and has been providing diabetes support and education to their members across the country. It was believed that their learnings and experience over the years will be essential in developing a program for this context |  |
