# Supplementary material for: Improving foot self-care in people with diabetes in Ghana: A development and feasibility randomised trial of a context appropriate, family-orientated diabetic footcare intervention
Source: PLoS One. 2024 May 8;19(5):e0302385. doi: 10.1371/journal.pone.0302385 (PMC11078378; doi:10.1371/journal.pone.0302385)
Supplement: S4 File — (DOCX) [file pone.0302385.s004.docx]

**Interview Topic Guide**

**Title of study: Nurse-led** Family-oriented Diabetic Foot Self-care Programme in Ghana; A Feasibility Randomised Controlled Trial with nested qualitative interviews at Komfo Anokye Teaching Hospital.

**Instructions**

- Explain the objectives of this study to interviewee
- Be sure that informed consent has being obtained for the interview and audio recording
- The interview guide is divided into three sections; A, B and C. Section A is for participants with diabetes, section B is for caregivers of persons with diabetes and section C is for Nurse participants. Use the appropriate section for each participant.

**Interview topic Guide**

**Section A: For participants with diabetes**

1. Please tell me about your feelings/impressions about the foot care training and education programme?

**PROMPTS:** components (Content, duration, delivery process by the nurse, timing, engagement/participation, venue, resources/logistics, applicability in everyday life)

1. Please tell me about what you have understood from the training and education programme?

**PROMPTS:** selfcare (foot care, knowledge/awareness about foot disease, care skills). How different is it from what you knew?

1. What outcomes or benefits will you expect to/likely to get from this and similar foot care programmes? What will you like to know concerning prevention of diabetes foot ulcer?

**PROMPTS:** awareness or knowledge on …, skills, behaviour, peer or family supports?

1. Generally, what will you say about the questionnaires/outcomes that you completed

**PROMPTS:** understanding (language, clarity), significance or relevance to you, appropriateness?

1. How will you describe your expectations or experience about this programme?

**PROMPTS**: Satisfaction with…, willingness to participate in similar future programmes, recommendation for others?

1. Anything else you want to say about this programme and future research?

**End of Interview for patients**

**Section B: For Family Caregiver Participants**

1. Please tell me about what you do to support your relative as a caregiver in a typical day.

**PROMPTS**: Diet planning/cooking, assisting with diabetes care, workload, difficulties, facilitators

1. Please tell me about your feelings/impressions about the foot care training and education programme received?

**PROMPTS**: components (Content, time duration, delivery process by the nurse, timing, engagement/participation, venue, resources/logistics, applicability in everyday life)

1. Generally, what will you say about the questionnaires/outcomes that you completed? **PROMPTS:** understanding (language, clarity), significance or relevance to you or appropriateness?
2. How will you describe your expectations about this foot care education programme? **PROMPTS**: Satisfaction with…, willingness to participate in such future programmes, recommendation for others?
3. What do you think about being a caregiver? **PROMPTS**: Lifestyle changes/ tell me more about that, anything else? How do you feel about that?
4. Anything else you want to say about this programme and future research?

**End of Interview for caregivers**

**Section C: Nurse participants**

1. Please tell me about your feelings/impressions about the foot care training and education programme?

**PROMPTS:** components (Content, time duration, delivery process by the nurse, timing, engagement/participation, venue, resources/logistics, applicability of the programme in everyday life)

1. What do you think about organising this and similar foot care training/education programme in your practice area/hospital?

**PROMPTS**: Facilitators/enablers, barriers/challenges

1. Anything else you want to say about this programme and future research?

**End of Interview for Nurse**

**THANK YOU FOR PARTICIPATING**
