# Supplementary material for: Improving foot self-care in people with diabetes in Ghana: A development and feasibility randomised trial of a context appropriate, family-orientated diabetic footcare intervention
Source: PLoS One. 2024 May 8;19(5):e0302385. doi: 10.1371/journal.pone.0302385 (PMC11078378; doi:10.1371/journal.pone.0302385)
Supplement: S5 File — (DOCX) [file pone.0302385.s005.docx]

S5: Intervention curriculum and strategies

| Week | Topic/duration | Content of sessions | Components of self-efficacy learning being use and delivery methods |
| --- | --- | --- | --- |
| **1** | Topics:   1. Diabetes and the Diabetic foot 2. Family Caregiver roles and support in foot care   Duration: 2hours | - Programme introduction (overview) - Diabetes education (definition, causes/risk factors, treatment and self-management, diabetes as a manageable chronic disease, overview of complications) - Providing information on how the feet is affected by diabetes. - Teaching on awareness of serious foot problems - Family caregiver role in prevention of foot problems - Discussion and teaching on caregiver roles and responsibilities. - Discussion facilitators and barriers amid family involvement in foot ulcer prevention - Discussion problem-solving strategies to deal with foot problems. - Motivating caregiver to actively support in foot check activities | Intensive health education  Verbal persuasion  Peer learning |
| **2** | Topic:   1. Diabetic footcare activities   Duration: 2hours | - Recap of family caregiver roles from the previous session. - Teaching on daily foot self-care activities and monitoring: - Daily foot inspection for foot problems - Daily washing and drying of feet. - Use of moisturiser - Massaging foot and foot exercise - Footwear choices - Nail care and when to seek care from a health professional. - Foot sensitivity checking - Annual foot examination by health professional | Intensive health education  Verbal persuasion and encouragement  Identifying and adjusting to stress and harnessing the support of social environment (caregiver)  Problem-solving  Peer learning |
| **3** | Topic:  Experiential workshop, skills training on footcare  Duration: 2hours | Skills demonstrating and role play on how to carry out foot checks at home:   - Physical feet inspection for problems detection - Use of mirrors to check bottom of feet. - Application of feet moisturizers - Use of proper footwear - Demonstration with samples of shoes and socks - Use of nail clippers to cut nails. - Use of 10g monofilament to check for protective feet sensation. - Return demonstrating of skills by study participants | Mastery experiences  Vicarious learning  Verbal encouragement, and feedback.  Skills training/Discussion |
| **4** | Topic: Reinforcement of foot education and skills training  Duration 2hours | - Recap of the various foot care activities taught in week 2. - Skill demonstrating and role play of various skills learnt in week 3. - Questions and answer session to clarify any doubt. - Refreshment of participants to conclude the programme | Skills training/Discussion  Vicarious learning/verbal encouragement |
