# Supplementary material for: Improving foot self-care in people with diabetes in Ghana: A development and feasibility randomised trial of a context appropriate, family-orientated diabetic footcare intervention
Source: PLoS One. 2024 May 8;19(5):e0302385. doi: 10.1371/journal.pone.0302385 (PMC11078378; doi:10.1371/journal.pone.0302385)
Supplement: S6 File — (DOCX) [file pone.0302385.s006.docx]

Coding of interviews:

| **Theme** | **Subthemes and supporting codes from patients and caregivers** | **Subtheme and supporting codes from Nurses** |
| --- | --- | --- |
| **Content of intervention** | **Proactive and interesting: “**the demonstration aspects of the program were so interactive and engaging. It appeared as if we were playing but learning a lot of things I didn’t know… I would like to come again’’ (P4 with diabetes)  **Provides relevant and new knowledge:** “We were taught how to check our feet and wash our feet. I used to wash my feet but not drying between my toes like we were taught yesterday. This is very good to know’’ (P3 with diabetes)  **Easy to understand:** “My mother cannot read because she not been to school... but because now you are using many photos and hands-on demonstration it is easy to learn” (P1 caregiver)  **Boosts confidence:** “with the personal practice at the program and observing others do it, I feel so capable and good ..., I hope when I go home, I will be able to continue” (P3 with diabetes) | **Engaging for patients and staff**: “We have received similar foot checks training in the past, but it was not engaging like this one that we are provided with the foot care items. This is really good information for us and the patients as well’ (Nurse P2)  **Easy to teach and understand:** “With the footcare items provided, it makes the teaching of patients easier and practical... if it were to be only talking, the patients easily forget when they get back home’ (Nurse P3) |
| **Delivery issues** | **Remote delivery preferred**: “I wish there was another way I could learn these things without missing my own work. Can it be done in the radio or television… or make sessions brief that we can finish and still go to market?’’ (Caregiver P 3).  **Non suitable venue:** ‘’… others like me cannot walk on these stairs because of my knee pains. ... I don’t know if you have another place we can do this education program?’’ (P3 with diabetes)  **Face-to-face and group delivery helpful**: “... you see, because we are all in the room, he (intervention provider) answered all my questions... and after I observed the other two people performed the foot checks, I was able to do it too” (P2 with diabetes) | **Face-to-face delivery possible only on specific days:** “... because of the time involved, meeting to provide the training for patients may be possible only on Thursday or Monday ... these days not always busy at the clinic. Unlike Tuesdays and Wednesday that the clinic is very heavily attended...” (nurse P2)  **Increases workload: ‘…**You see the skills demonstration and return demonstration is very good for them (patients) to learn but it increases our workload ... and we the nurses are few compared to our workload’ (nurse P4) |
| **Duration and frequency of intervention** | **Intervention session too long**: “...because I need to go to work ... ‘’ the program very good but the sessions were too long… maybe you can make each day very short only 1hour in the morning…over a period’’ (P2 Caregiver) | **Intervention time should be reduced:** “...a lot of things that needs doing really ... but not all can be done. We are constraint in so many ways but is worth preserving small time for patient education like this... lets work at making the program very brief to be delivered within shortest possible time” (nurse p4) |
| **Acceptability of intervention** | **Importance of intervention for health:** “I like it...very good program,.. my mother diet after amputation because of diabetes ... may be it could have been prevented if programs like this were available” (P4 Caregiver)  “This my second time participating in research ... but always after the research they don’t continue the programs at the hospital ... This is very good education that should be continued by the diabetes clinic authorities after this research” (P 3 with diabetes)  **Is a moral responsibility**: “it is important to know these things at home... when I am old, my children will also take care of me ... so I have to come and learn to be able to assist her now that she old ... it is my duty” (P2 caregiver) | **A needed intervention to prevent foot disease**: “Diabetic foot disease is really on the increase; we see them in the clinic every day and so this project seeking to address this is a good idea must be given all the attention and support’’ (nurse P3)  **Provides valuable skills**: “I am not a diabetes nurse specialist ... it is from programs like this that we learn ... is a good idea....” (Nurse P5) |
| **Caregiver requirement** | **Excludes people:** ‘’… I have a friend that I know she will be very interested in this program, but she lives alone... can you consider her to participate? ... because all her children are grown and working outside Kumasi” (P1 with diabetes).  **“...**My son goes to work in the morning and only comes home in the evening to assist me ... if only you could teach us those with diabetes, we can also inform our children and those at home what to do. ... he (referring to his son) is happy to help me but very busy with work” (P3 with diabetes) | **Difficulty getting caregivers**: “‘…Is it possible to do it for only patients, because even though the carers are very important in this process, some patients really are not able to bring their caregivers’ (nurse P 2). |

**CFIR - Barriers and facilitators to the intervention**

| **CFIR domain** | **Element of CFIR** | **Facilitator** | **Participant Verbatim Quote** |
| --- | --- | --- | --- |
| Intervention Characteristic | Relative advantage | It is easier to teach patients using equipment | ‘…With the footcare items provided, it makes the teaching of patients easier and practical... if it were to be only talking, the patients easily forget when they get back home’ (Nurse P3) |
|  | Design quality and packaging | Involving carers for older people | ‘…. The idea of involving the caregivers is great especially for the aged and those with bad eyesight... and because there is skill demonstration everyone who cannot read will still understand...’ (Nurse P4) |
| Inner setting | Relative priority | The need to curb the incidence of foot disease | ‘’...the number of limb amputations we record every year and the bad open wounds we see in this clinic is not just acceptable, authorities need to do something ... programs like this may help’’ (Nurse P1) |
|  | Compatibility | Nurses have previously been given similar foot checks training. | ‘We have received similar foot checks training in the past, but it was not intensive like this one that we are provided with the foot care items. This is really good information for us and the patients as well’ (Nurse P2) |
|  | Leadership engagement | Available supervisors | ‘’…is alright once there is somebody supervising and if there is anything I am not sure, I can ask for clarification… so is okay to conduct future studies like this and make the practice part of our routine’’ (Nurse P3) |
| Characteristics of individuals | Self-efficacy | Increased self-confidence | ‘’I think I felt more confident delivering the training after the first session…I was now used to the steps ... ’’ (Nurse P4) |
|  | Other personal attributes | Readiness to learn the intervention | ‘’I am not a diabetes specialist just like most of my colleagues, it is from programs like this that we learn, it may be a good idea if we are taken for specialist training… delivering such an intervention wouldn’t have been a problem… but we will learn it’’ (nurse P5) |
|  | Executing | Supervision from researcher and/or clinic managers | ‘’…you see, doing these things are good but we will always need a senior staff to be around like the way our in charge (referring to unit manager) has been doing... because you know we are not diabetes specialist ...” (Nurse p4) |
| **CFIR domain** | **Element of CFIR** | **Barrier** | **Verbatim Quote** |
| Intervention Characteristic | Design quality and packaging | Difficulty in getting caregivers to participate | ‘’Most persons will be interested in this intervention program, but they will not be able to get the caregivers to come with them, … is good, but these days all carers are working … only supporting when they are back home with their sick relatives’’ (Nurse P3) |
|  | Complexity | Intervention sessions are too long | ‘…Because it is research many of us stayed till the end even though it took longer than I expected ... In actual practice we may not have all this time regularly’ (nurse P2) |
| Inner setting | Relative priority | Nurses and intervention providers have multiple competing roles | ‘I usually do a lot of different things like supervision of other staff/students and taking care of patients, so sometimes it is difficult to take part in research programs…’ (Nurse p5) |
|  | Available resources | Limited resources especially at the district and subdistrict level | ‘Hmmm sometimes is not like you cannot do it ooo but the resources needed will not always be available. Here may even be better than district and sub-district levels are worst’ (Nurse P3) |
| Characteristics of individuals | Self-efficacy | Lack of diabetes specialist knowledge | ‘’... these things we know them ... but sometimes you are just afraid to volunteer and teach it ... because you are not a specialist and the hospital hasn’t taken you for any training ..’’ (Nurse P4) |
| Process | Executing | Increase in workload | ‘’…Is a good program but sometimes we are only five nurses on duty and have to attend to over 80 patients hmm, so you don’t get the time to teach them all these things’’. |
