# Supplementary material for: Improving foot self-care in people with diabetes in Ghana: A development and feasibility randomised trial of a context appropriate, family-orientated diabetic footcare intervention
Source: PLoS One. 2024 May 8;19(5):e0302385. doi: 10.1371/journal.pone.0302385 (PMC11078378; doi:10.1371/journal.pone.0302385)
Supplement: Attachment — Submitted filename: Response to Editor.docx [file pone.0302385.s008.docx]

Professor Sascha Köpke

Academic Editor

PLOS ONE

10^th^ November 2023

Dear Professor Sascha Köpke,

Thank you so much for the review and signposting to the resources. That has been very helpful in improving the paper. We have now used the correct reporting guidelines, that is the CONSORT extension for pilot and feasibility trials. We have added some additional text to the paper to adequately reflect the filled checklist. Details of the randomisation process has been added to make it clearer. Also, the sequence of some subheading such as participants, recruitment and randomisation have been reorganised to make the paper easier to follow.

The trial registration link has been added together with the registration number.

We hope you find our manuscript suitable for publication and look forward to hearing from you in due course.

Thank you,

Joseph Suglo

Florence Nightingale Faculty of Nursing, Midwifery & Palliative Care

King’s College London

James Clerk Maxwell Building 1.32

London
