# Supplementary material for: Improving foot self-care in people with diabetes in Ghana: A development and feasibility randomised trial of a context appropriate, family-orientated diabetic footcare intervention
Source: PLoS One. 2024 May 8;19(5):e0302385. doi: 10.1371/journal.pone.0302385 (PMC11078378; doi:10.1371/journal.pone.0302385)
Supplement: Attachment — Submitted filename: Response to the reviewers.docx [file pone.0302385.s009.docx]

The Academic Editor

PLOS ONE

7^th^ February 2024

Dear Professor Sascha Köpke,

We would like to express our sincere gratitude to you and the reviewers for your thoughtful and constructive feedback on our manuscript entitled "Improving foot self-care in people with diabetes in Ghana: A development and feasibility randomised trial of a context appropriate, family-orientated diabetic footcare intervention”.

We have carefully considered the reviewers' comments and have made revisions to address their concerns. In the table below, we provide detailed responses to each of the reviewers' comments.

Thank you once again for your valuable feedback and support.

Joseph Suglo

Florence Nightingale Faculty of Nursing, Midwifery & Palliative Care

King’s College London

James Clerk Maxwell Building 1.32

London

Table 1: Response to the reviewers

| Reviewer | comments | Authors response | Location within revised paper |
| --- | --- | --- | --- |
| Editor | The abstracts need revision, please make sure to transparently report numbers here, i.e. numbers of participants and numbers (together with 95% CIs) for main results, if applicable. | We acknowledge the importance of transparently reporting numerical data, including the numbers of participants and main results with their corresponding 95% confidence intervals in the abstract. In our revised version, we have ensured that these essential details are prominently reported. | Page 2 |
| Reviewer 1 | This manuscript presents analysis of data generated from a feasibility randomized trial to determine the effectiveness of a diabetic footcare intervention (wrt. a control), in a diabetic population in Ghana. The trial was approved by the respective ethics board, and registered within the PACT registry. The study objectives are on target. However, I mostly have some concerns/comments in the statistical design and analytical framework, which may require attention. | We appreciate the opportunity to address your concerns regarding the statistical design and analytical framework |  |
| Reviewer 1 | Sample size/power: The sample size/power statement provided is not adequate; although this is a feasibility trial, some thoughtful presentation of the anticipated sample size is required. For example, please read: https://www.ncbi.nlm.nih.gov/pmc/articles/PMC8849521/ | Thanks so much for the review and signposting to a very valuable resource paper. Very insightful reading that paper. We have now added some more details and rational for our sample size. Small sample size in this feasibility trial has also been discuss as part of the study limitations | Page 8 lines 154-167 and page 31 line 399 |
| Reviewer 1 | There should be clear mention of the statistical test used (1-, or 2-sided), the level of significance, and the desired effect size the authors like to see. | Thank for the constructive review comments which helps to strengthen our paper. We ensured that the details are provided under a separate heading: Statistical analysis. | Page 14 lines 284 - 300 |
| Reviewer 1 | Create a separate subheading | Thank you. New heading introduced | Page 14 |
| Reviewer 1 | Statistical analysis plan: Independent sample t-tests were proposed, which is only valid under assumptions of Normality. Alternative (nonparametric) tests, under violations of Gaussian assumptions, were not mentioned, such as the Wilcoxon rank-sum test. Also, have the authors assessed Gaussian/Normal assumptions of the quantities to be compared via t-tests? | Thank for the constructive review comments which helps to strengthen our paper. We ensured that the details are provided under a separate heading: Statistical analysis. | Page 14 lines 284 - 300 |
| Reviewer 1 | Missing Data: How is missing data handled in statistical analysis? | Thank you for noting this. We have now indicated how missing data were handle | Page 15 lines 296-300 |
| Reviewer 1 | Results: At various places, results are stated with estimates, and 95% confidence intervals. While this is nice, adding the p-values would be more relevant. | Thanks so much for the observation and suggestion. We chose not to present the p-values as the study was not powered to estimate the effect of the intervention. We have discussed the small sample size, wide confidence intervals and non-presentation of p values in our discussion and in our study’s limitation | Page 29 & 31 |
| Reviewer 1 | Discussion Section: This section should clearly allude to future studies on other populations and geographical regions to further validate the current findings. The findings from this protocol will only be limited to this population from Ghana. | Your review very much appreciated as it helps to strengthen the value of our paper. Evidence of the relevance of this intervention to other populations with strong familial bonds has now been included. | Page 31 lines 388 - 395 |
| Reviewer 2 | I think the study is well written and all comments raised by previous authors have been adequately addressed. | Thank you. |  |
| Reviewer 2 | Abstract: In the abstract, you did not mention the control group the intervention was compared to. It would be appropriate to include it in the abstract. | Thanks for noting this. The usual clinic care as control group has now been indicated in the abstract and its detail description provided in the manuscript | Page 2, lines 24-28 |
| Reviewer 2 | Introduction: As indicated by the authors, the family-based intervention has already being explored or used by other investigators in LMIC setting and has demonstrated improved outcomes. Has this intervention being used to improve patients outcomes in Ghana?. If yes, how will your study be different from the previously existing studies. | We appreciate your review comments. The intervention has not been evaluated anywhere in Africa. We have added few words in the introduction to make this clearer. | Page 5, lines 84-89 |
| Reviewer 2 | Methods: Line 104: Can you briefly state or describe the evidence-based intervention identified in the previous systematic review? | Thank you for the review. A brief of the evidence-based intervention has been indicated and reference to full intervention provided | Page 6, lines 106-111 |
